# Supplementary material for: Loss of FCoV-23 spike domain 0 enhances fusogenicity and entry kinetics
Source: Nature. 2025 Jul 9;645(8079):235–43. doi: 10.1038/s41586-025-09155-z (PMC12408340; doi:10.1038/s41586-025-09155-z)

nature portfolio

https://doi.org/10.1038/s41586-025-09155-z

Supplementary information

Loss of FCoV-23 spike domain 0 enhances fusogenicity and entry kinetics

In the format provided by the authors and unedited

Supplementary information guide

Supplementary Figures 1-13. Uncropped SDS-PAGE and western blots of the indicated figures.

Supplementary Table 1. Cryo-EM data collection, refinement and validation statistics.

Supplementary Table 2. Biolayer interferometry kinetic parameters for binding of *Fc*APN and *Cf*APN to immobilized FCoV-23 RBD and CCoV-HuPn-18 RBD.

SI Figure 1

Raw image

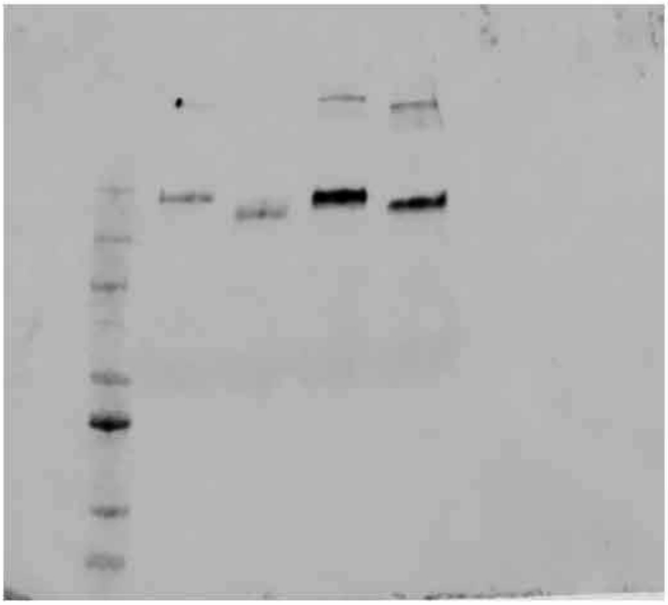

Extended data Figure 1h

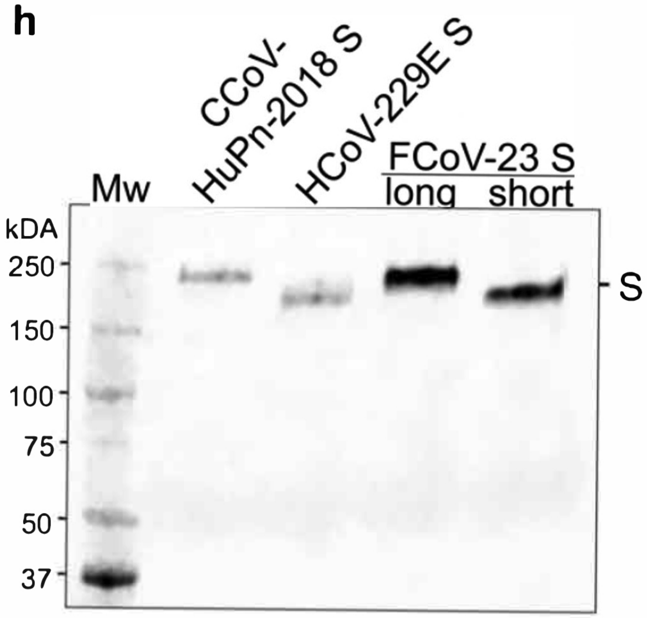

SI Figure 2

Raw image 4a, 4d

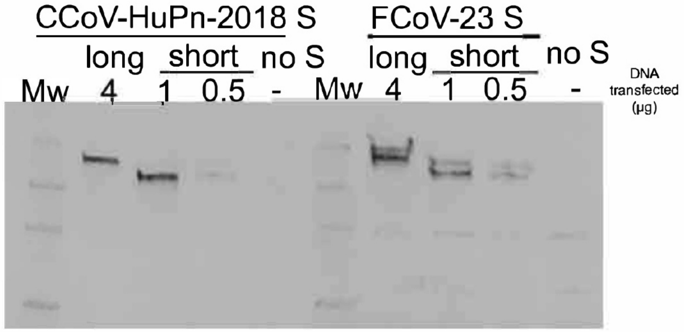

Extended data Figure 4a

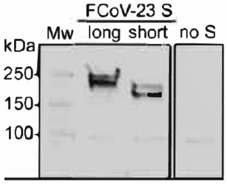

Extended data Figure 4d

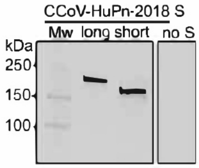

SI Figure 3

Raw image 4b

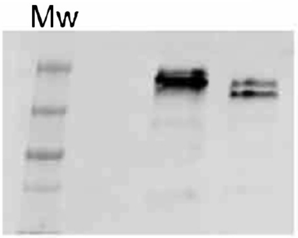

Extended data Figure 4b

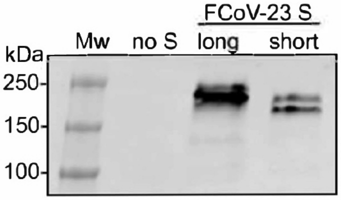

SI Figure 4

Raw image 4e

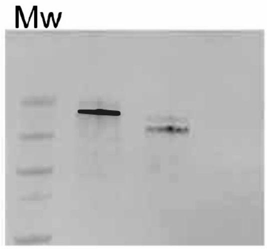

Extended data Figure 4e

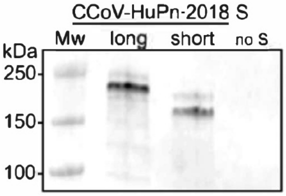

SI Figure 5

Raw image 4c

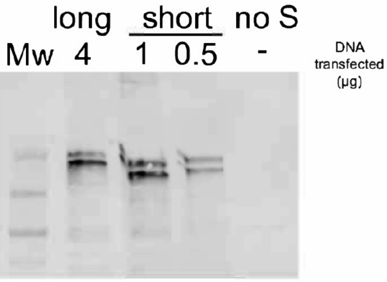

Extended data Figure 4c

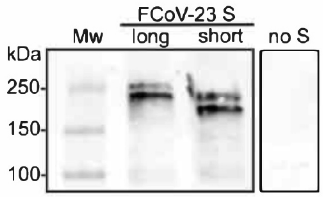

Raw images

SI Figure 6

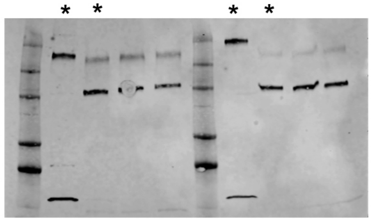

*lanes used in the figure

SI Figure 7

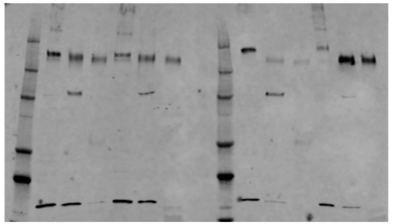

SI Figure 8

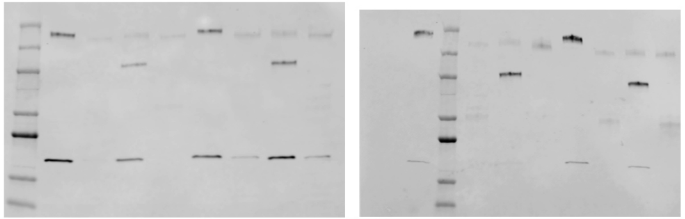

Extended data Figure 5 g, h, i

g **VSV FCoV-23 S**

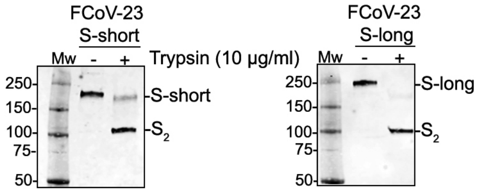

h **VSV FCoV-23 S + *Cf*APN**

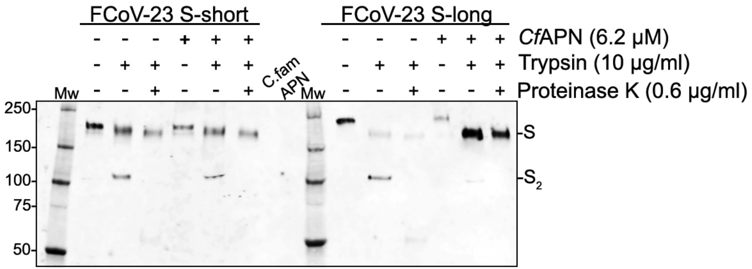

i **VSV-FCoV-23 S + VSV-*Cf*APN**

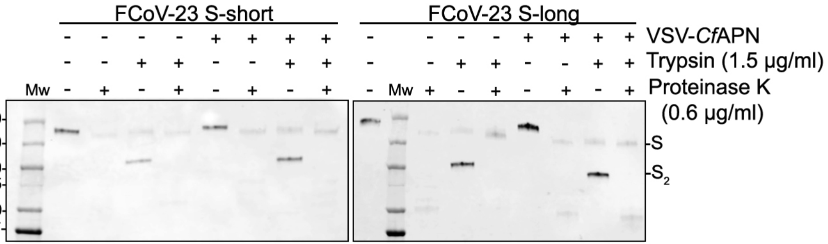

SI Figure 9

Raw image

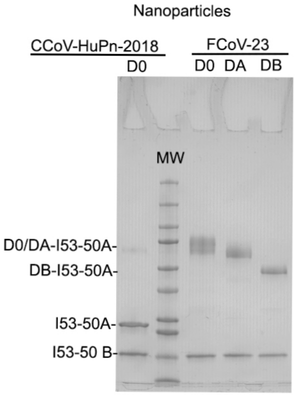

Extended data Figure 6b

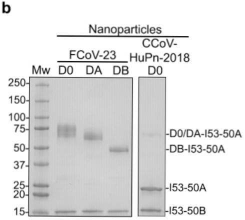

SI Figure 10 Raw images

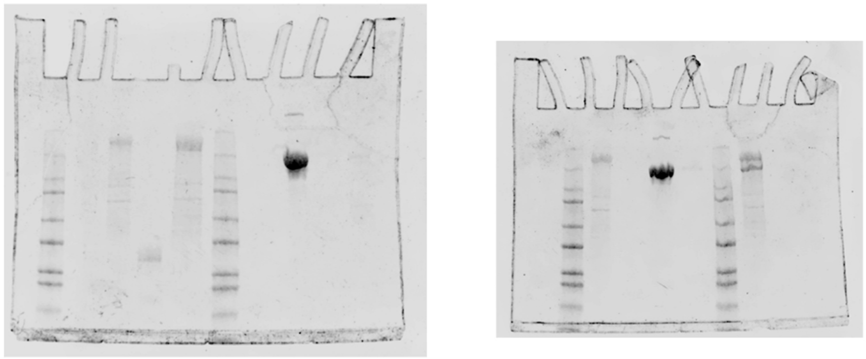

Extended data Figure7c

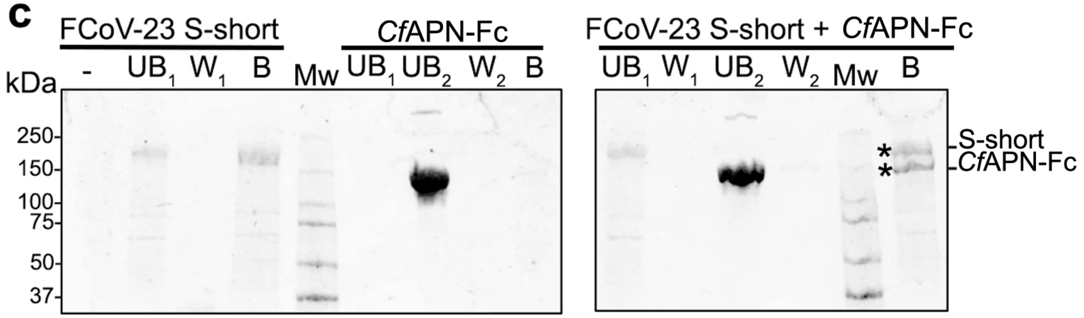

SI Figure 11 Raw images

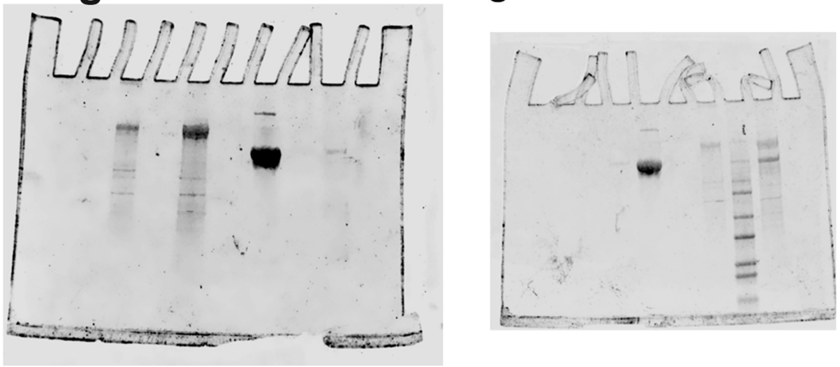

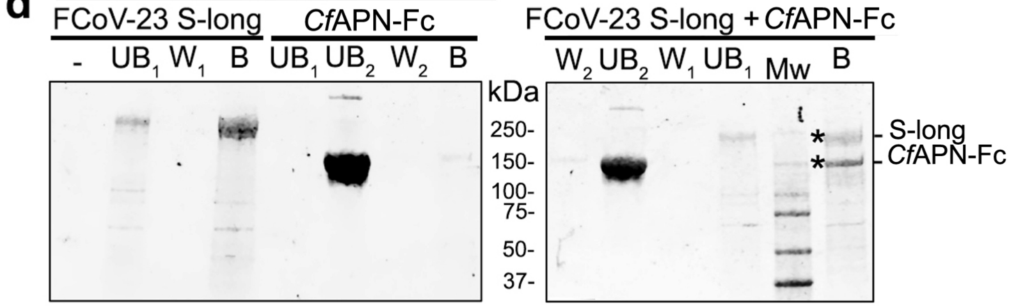

SI Figure 12

Raw images

* lanes used in the figure

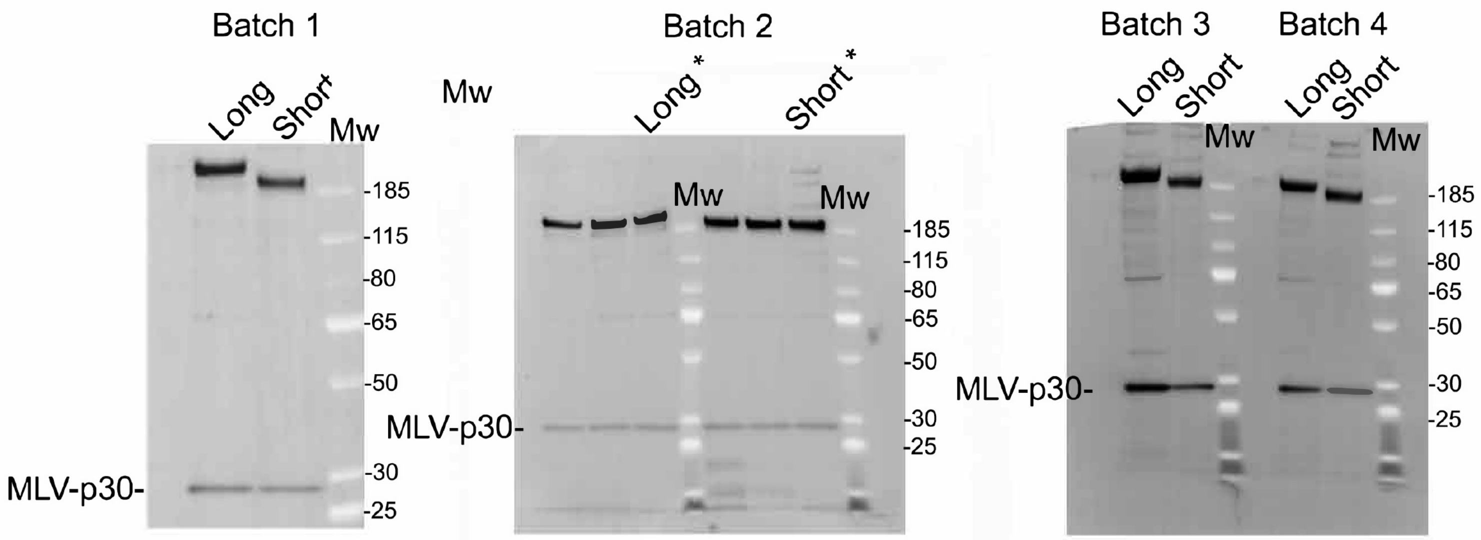

Extended data Figure 9c

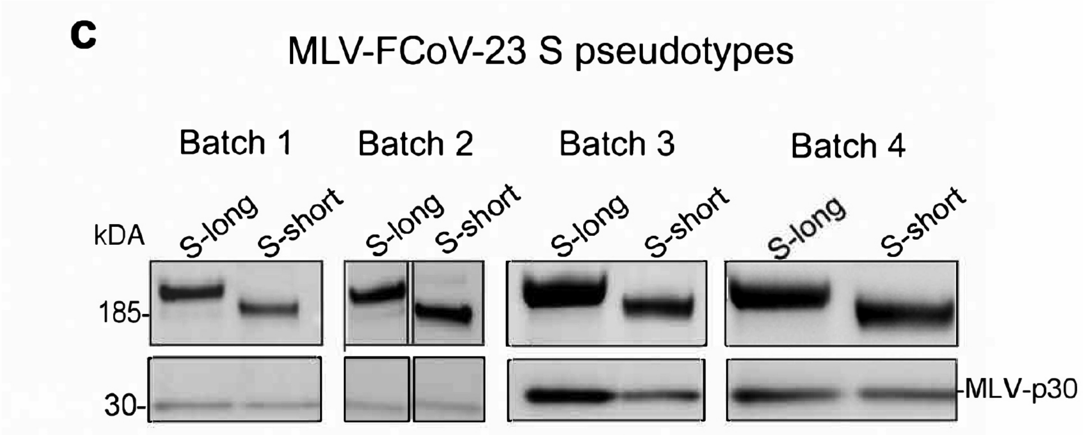

SI Figure 13

Raw images

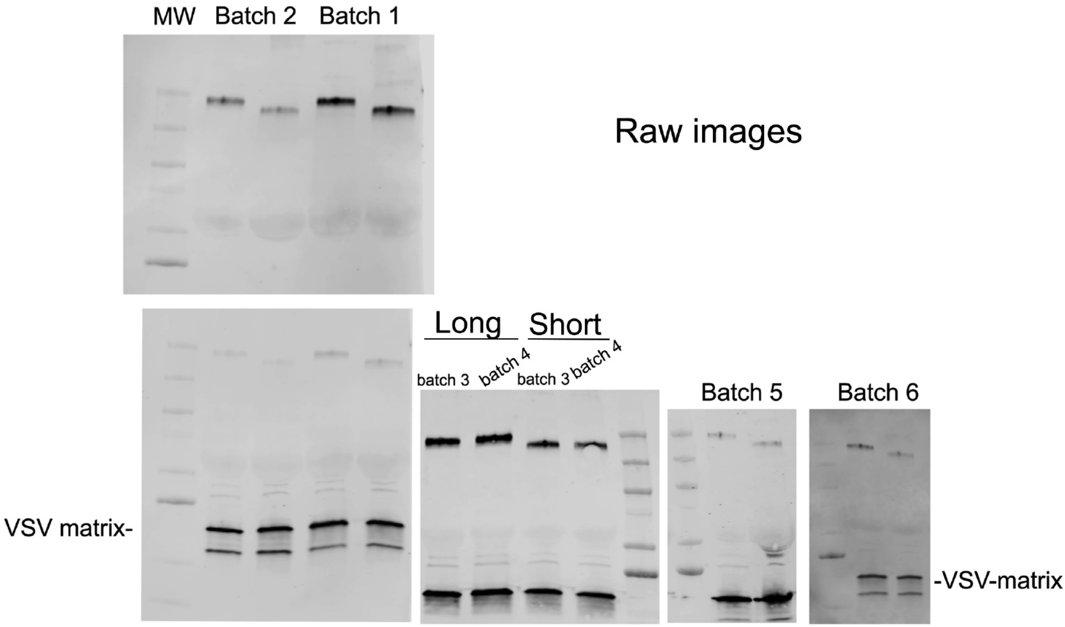

Extended data Figure 10

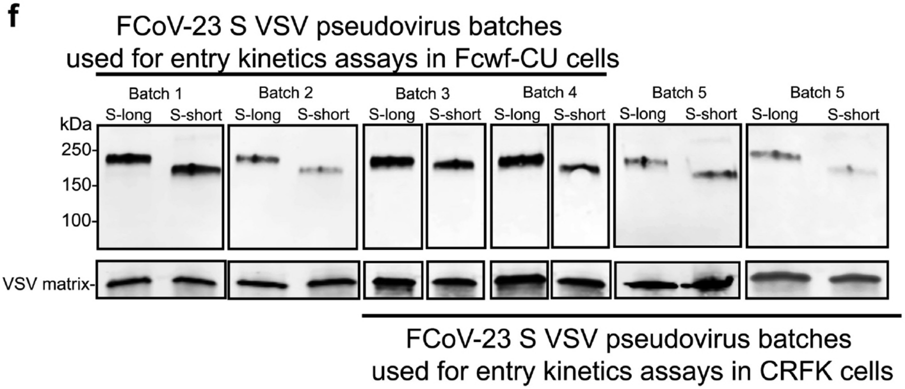

Supplement: Supplementary file 1 — This file contains Supplementary Figs. 1–13 and Supplementary Tables 1 and 2. [file 41586_2025_9155_MOESM1_ESM.pdf]
