# Supplementary material for: Loss of FCoV-23 spike domain 0 enhances fusogenicity and entry kinetics
Source: Nature. 2025 Jul 9;645(8079):235–43. doi: 10.1038/s41586-025-09155-z (PMC12408340; doi:10.1038/s41586-025-09155-z)
Supplement: Supplementary file 2 — Reporting Summary [file 41586_2025_9155_MOESM2_ESM.pdf]

nature portfolio

Corresponding author(s): David Veesler

Last updated by author(s): 2/12/2025

Reporting Summary

Nature Portfolio wishes to improve the reproducibility of the work that we publish. This form provides structure for consistency and transparency in reporting. For further information on Nature Portfolio policies, see our Editorial Policies and the Editorial Policy Checklist.

Statistics

For all statistical analyses, confirm that the following items are present in the figure legend, table legend, main text, or Methods section.

n/a	Confirmed	
☐	☒	The exact sample size (*n*) for each experimental group/condition, given as a discrete number and unit of measurement
☐	☒	A statement on whether measurements were taken from distinct samples or whether the same sample was measured repeatedly
☐	☒	The statistical test(s) used AND whether they are one- or two-sided<br>*Only common tests should be described solely by name; describe more complex techniques in the Methods section.*
☒	☐	A description of all covariates tested
☒	☐	A description of any assumptions or corrections, such as tests of normality and adjustment for multiple comparisons
☐	☒	A full description of the statistical parameters including central tendency (e.g. means) or other basic estimates (e.g. regression coefficient) AND variation (e.g. standard deviation) or associated estimates of uncertainty (e.g. confidence intervals)
☒	☐	For null hypothesis testing, the test statistic (e.g. F, t, r) with confidence intervals, effect sizes, degrees of freedom and P value noted<br>*Give P values as exact values whenever suitable.*
☒	☐	For Bayesian analysis, information on the choice of priors and Markov chain Monte Carlo settings
☒	☐	For hierarchical and complex designs, identification of the appropriate level for tests and full reporting of outcomes
☒	☐	Estimates of effect sizes (e.g. Cohen's d, Pearson's r), indicating how they were calculated

Our web collection on statistics for biologists contains articles on many of the points above.

Software and code

Policy information about availability of computer code

Data collection	Datasets were acquired using a FEI Titan Krios transmission electron microscope operated at 300 kV equipped with a Gatan K3 direct detector and a Gatan Quantum GIF energy filter, operated with a slit width of 20eV. Automated data collection was carried out using the Leginon software at a nominal magnification of 105,000x corresponding to a pixel size of 0.843 A.
Data analysis	CryoEM model building and analysis<br>Model Angelo114 was used to generate an initial model and UCSF Chimera 1.8 115 and Coot 0.9.8.8116 were used to manually build the model. Model was refined and rebuilt into the maps using Coot 0.9.8.8, Phenix 1.21117 and Rosetta 2021.07.61567118,119. Model validation was done using Molprobity120 and Privateer121 from the CCP4i2 suite. Figures were generated using UCSF ChimeraX 1.8122. Palmitoleic acids resolved in our cryo-EM maps were built in the final models based on previous findings on PEDV.<br><br>VSV pseudotyped virus infections and neutralizations<br>Relative luciferase units were plotted and normalized in Graphpad Prism 10<br><br>Biolayer interferometry (BLI) binding assays<br>Data were baseline subtracted, and the plots were fitted using the Sartorius analysis software (v.11.1). Data were plotted in Graphpad Prism 10.

For manuscripts utilizing custom algorithms or software that are central to the research but not yet described in published literature, software must be made available to editors and reviewers. We strongly encourage code deposition in a community repository (e.g. GitHub). See the Nature Portfolio guidelines for submitting code & software for further information.

Data

Policy information about availability of data

All manuscripts must include a data availability statement. This statement should provide the following information, where applicable:

- Accession codes, unique identifiers, or web links for publicly available datasets
- A description of any restrictions on data availability
- For clinical datasets or third party data, please ensure that the statement adheres to our policy

All data supporting the findings of this study are available within the paper and its Supplementary Information.
Accession codes for the structures presented in this work are: EMD-46714/PDB 9DB3 for FCoV-23 S-long with swung-out D0 (global refinement), EMD-46739/PDB 9DBZ for FCoV-23 S-long with mixed D0 conformations (global refinement), EMD-46716/PDB 9DBE for the local refinement of D0 from the FCoV-23 S-long swung-out dataset, EMD-46710/PDB 9DB1 for the local refinement of the proximal D0 from the FCoV-23 S-long with mixed D0 conformations, EMD-46709 PDB 9DB0 for FCoV-23 S-short, and EMD-46708/PDB 9DAZ for the F.cat APN-bound FCoV-23 RBD.

Research involving human participants, their data, or biological material

Policy information about studies with human participants or human data. See also policy information about sex, gender (identity/presentation), and sexual orientation and race, ethnicity and racism.

Reporting on sex and gender	N/A
Reporting on race, ethnicity, or other socially relevant groupings	N/A
Population characteristics	N/A
Recruitment	N/A
Ethics oversight	N/A

Note that full information on the approval of the study protocol must also be provided in the manuscript.

Field-specific reporting

Please select the one below that is the best fit for your research. If you are not sure, read the appropriate sections before making your selection.

☒ Life sciences ☐ Behavioural & social sciences ☐ Ecological, evolutionary & environmental sciences

For a reference copy of the document with all sections, see nature.com/documents/nr-reporting-summary-flat.pdf

Life sciences study design

All studies must disclose on these points even when the disclosure is negative.

Sample size	We use 10 mice to generate sera against HCoV-229E S (a typical group size)
Data exclusions	We did not exclude any data of the analysis
Replication	All binding and pseudovirus entry/neutralization experiments were replicated with at least two independent biological replicates, as indicated.
Randomization	We did not need to randomize the mice since we used this study only to generate polyclonal antibodies.
Blinding	We did not need to randomize the mice since we used this study only to generate polyclonal antibodies and all animals were analyzed.

Reporting for specific materials, systems and methods

We require information from authors about some types of materials, experimental systems and methods used in many studies. Here, indicate whether each material, system or method listed is relevant to your study. If you are not sure if a list item applies to your research, read the appropriate section before selecting a response.

Materials & experimental systems

n/a	Involved in the study
☐	☒ Antibodies
☐	☒ Eukaryotic cell lines
☒	☐ Palaeontology and archaeology
☐	☒ Animals and other organisms
☒	☐ Clinical data
☒	☐ Dual use research of concern
☒	☐ Plants

Methods

n/a	Involved in the study
☒	☐ ChIP-seq
☒	☐ Flow cytometry
☒	☐ MRI-based neuroimaging

Antibodies

Antibodies used

76El monoclonal antibody (in house generated), HA tag monoclonal antibody (Thermofisher, cat# 26183, lot: YL384846), HA rabbit polyclonal antibody (Proteintech, cat# 51064-2, lot:00154837), anti-MLV p30 antibody (Abcam, cat# ab130757, lot: 1047279-23), , VSV-M antibody (Kerafast, cat # EB0011, lot: 200826), 488-conjugated goat antimouse
secondary antibody
(Thermofisher, cat# A-11029, lot: 2821059), Alexa Fluor 680-conjugated donkey anti-human secondary antibody (Jackson Immuno Research, code: 709-625-149, lot:161754), Alexa Fluor 680-conjugated goat antimouse
secondary antibody (Jackson
Immuno Research, code: 115-625-174, lot:161031), Alexa Fluor 680-conjugated goat anti-rabbit secondary antibody (Jackson Immuno Research, code: 111-625-144, lot:145147)

Validation

76El monoclonal antibody. Sun, X., Yi, C., Zhu, Y. et al. Neutralization mechanism of a human antibody with pan-coronavirus reactivity including SARS-CoV-2. Nat Microbial 7, 1063-1074 (2022). https://doi.org/10.1038/s41564-022-01155-3.

HA monoclonal antibody (Thermo Fisher, https://www.thermofisher.com/antibody/product/HA-Tag-Antibody-clone-2-2-2-14-Monoclonal/26183)

HA-tag polyclonal antibody (Proteintech,, https://www.ptglab.com/products/HA-tag-Antibody-51064-2-AP.htm?srsltid=AfmBOoord6gacBa-N5uOxqfAsVXqOhkiKQvY7YjHkboTCIKutsnQE3KN and publications https://www.ptglab.com/products/HA-tag-Antibody-51064-2-AP.htm?srsltid=AfmBOoord6gacBa-N5uOxqfAsVXqOhkiKQvY7YjHkboTCIKutsnQE3KN#publications)

VSV M (23H12) monoclonal antibody (Kerafast, file:///Users/tortoric/Desktop/EB0011.pdf)

MLV p30 antibody (Abcam, https://www.abcam.com/en-us/products/primary-antibodies/mlv-p30-antibody-4b2-ab130757)

488-conjugated goat anti-mouse secondary antibody (Thermo Fisher, https://www.thermofisher.com/antibody/product/Goat-anti-Mouse-IgG-H-L-Highly-Cross-Adsorbed-Secondary-Antibody-Polyclonal/A-11029)

Alexa Fluor 680-conjugated donkey anti-human secondary antibody (Jackson Immuno Research, https://www.jacksonimmuno.com/catalog/products/709-625-149)

Alexa Fluor 680-conjugated goat anti-rabbit (Jackson Immuno Research, https://www.jacksonimmuno.com/catalog/products/111-625-144)

Alexa Fluor 680-conjugated goat anti-mouse (Jackson Immuno Research, https://www.jacksonimmuno.com/catalog/products/115-625-174)

Eukaryotic cell lines

Policy information about cell lines and Sex and Gender in Research

Cell line source(s)

HEK293T (CRL-3216), A549 (CRM-CCL-185), Calu3 (HTB-55) LLC-MK2 (CCL-7), BHK- 21 (CCL-10), L2 (CCL-149),DF-1 (CRL-3586), CRFK (CCL-94), AK-0 (CCL-150), A-72 (CRL-1542), MOCK (CCL-34), LLC-PKl (CL-101), MDBK (CCL-22), av KID, Fcwf-CU, VeroE6-TMPRSS2 (JCRB1819). Cell lines ExpiCHO cells and Expi293F cells were obtained from ThermoFisher Scientific. Human bronchial epithelial cells (HBEC cells) were provided by Dr. Richard Cerione lab at Cornell University. Ovine kidney cells (OV KID) and Ovine fetal turbinate (OFT) cells were provided by Dr. Diego Diel at Cornell University Animal Health Diagnostic Center. Feline macrophage-like cells, Fcwf-4, were initially obtained from ATCC (CRL-2787) and their progeny (Fcwf-CU) was selected by Dr. Edward Dubovi and Dr. Gary Whittaker at Cornell University to be significantly better at propagating feline coronavirus.

Authentication

None of the cells were authenticated

Mycoplasma contamination

Cell lines were not routinely tested for mycoplasma contamination.

Commonly misidentified lines
(See ICLAC register)

No commonly misidentified lines were used in this study

Animals and other research organisms

Policy information about studies involving animals; ARRIVE guidelines recommended for reporting animal research, and Sex and Gender in Research

Laboratory animals	Female BALB/cAnNHsd mice were purchased from Envigo (order code 047) at 7 weeks of age and were maintained in a pathogen-free facility within the Department of Comparative Medicine at the University of Washington, Seattle, accredited by the Association for Assessment and Accreditation of Laboratory Animal Care (AAALAC).
Wild animals	No wild animals were used in this study
Reporting on sex	We did not analyze sex of the mice as a variable as it was not relevant to our study given that the mice were used only to generate polyclonal antibodies.
Field-collected samples	No field-collected samples were used in this study
Ethics oversight	Animal experiments were conducted in accordance with the University of Washington's Institutional Animal Care and Use Committee under protocol 4470-01

Note that full information on the approval of the study protocol must also be provided in the manuscript.

Plants

Seed stocks	N/A
Novel plant genotypes	N/A
Authentication	N/A
